# Investigating the Impact of Disrupting the Glutamine Metabolism Pathway on Ammonia Excretion in Crucian Carp (*Carassius auratus*) under Carbonate Alkaline Stress Using Metabolomics Techniques

**DOI:** 10.3390/antiox13020170

**Published:** 2024-01-29

**Authors:** Yanchun Sun, Chuanye Geng, Wenzhi Liu, Yingjie Liu, Lu Ding, Peng Wang

**Affiliations:** 1 Department of Food Science and Engineering, College of Food Science and Technology, Shanghai Ocean University, Shanghai 201306, China; gengchuanye1998@163.com (C.G.); liuwenzhi0457@163.com (W.L.); liuyj8756@163.com (Y.L.); dinglu1083@163.com (L.D.); 2 Laboratory of Quality & Safety Risk Assessment for Aquatic Products, Heilongjiang River Fisheries Research Institute of Chinese Academy of Fishery Sciences, Ministry of Agriculture and Rural Areas, Harbin 150070, China; wapg911@126.com

**Keywords:** crucian carp (*Carassius auratus*), carbonate alkaline stress, metabolomics, glutamate pathway, serum, ammonia excretion

## Abstract

With the gradual decline in freshwater resources, the space available for freshwater aquaculture is diminishing and the need to maximize saline water for aquaculture is increasing. This study aimed to elucidate the impact mechanisms of the disruption of the glutamate pathway on serum metabolism and ammonia excretion in crucian carp (*Carassius auratus*) under carbonate alkaline stress. A freshwater control group (C group), a 20 mmol/L NaHCO_3_ stress group (L group), and a 40 mmol/L NaHCO_3_ stress group (H group) were established. After 30 days of exposure, methionine sulfoximine (MSO) was injected to block the glutamate pathway metabolism, and the groups post-blocking were labeled as MC, ML, and MH. Ultra-high-performance liquid chromatography coupled with the quadrupole time-of-flight mass spectrometry (UPLC-Q-TOF/MS) metabolomics technique was employed to detect changes in the composition and content of crucian carp serum metabolites. Significant differential metabolites were identified, and related metabolic pathways were analyzed. The results revealed that, following the glutamate pathway blockade, a total of 228 differential metabolites (DMs) were identified in the three treatment groups. An enrichment analysis indicated significant involvement in glycerophospholipid metabolism, arachidonic acid metabolism, sphingolipid metabolism, purine metabolism, arginine and proline biosynthesis, pantothenate and CoA biosynthesis, glutathione metabolism, and fatty acid degradation, among other metabolic pathways. The results showed that ROS imbalances and L-arginine accumulation in crucian carp after the glutamate pathway blockade led to an increase in oxidative stress and inflammatory responses in vivo, which may cause damage to the structure and function of cell membranes. Crucian carp improves the body’s antioxidant capacity and regulates cellular homeostasis by activating glutathione metabolism and increasing the concentration of phosphatidylcholine (PC) analogs. Additionally, challenges such as aggravated ammonia excretion obstruction and disrupted energy metabolism were observed in crucian carp, with the upregulation of purine metabolism alleviating ammonia toxicity and maintaining energy homeostasis through pantothenate and CoA biosynthesis as well as fatty acid degradation. This study elucidated the metabolic changes in crucian carp under carbonate alkaline stress after a glutamate pathway blockade at the cellular metabolism level and screened out the key metabolic pathways, which provide a scientific basis for further in-depth studies on the ammonia excretion of freshwater scleractinian fishes under saline and alkaline habitats at a later stage.

## 1. Introduction

In various regions worldwide, particularly in Central and East Asia, saline–alkaline waters are extensively distributed, with China alone encompassing an area of approximately 9.9 × 10^7^ hm^2^. The majority of these areas remain biologically barren and have not been effectively utilized [1]. Saline–alkaline water types are primarily categorized as sulfate, chloride, and carbonate. Sulphate water is mainly composed of compounds of sulphate ions (SO_4_^2−^) and other metal ions, which usually have high levels of acidity, and its toxicity to aquatic organisms is mainly manifested in the damage to gill tissues and the inhibition of enzyme activities [2]. The main cations in chloride are sodium and potassium ions, and the anion is chloride, which may affect the osmotic balance and metabolic activities of aquatic organisms at certain concentrations [3]. Among these, carbonate-dominated areas are the most expansive, characterized by elevated levels of electrical conductivity, pH, and carbonate alkalinity (CA), as well as by a poor water buffering capacity [4]. The high alkalinity of the water can disrupt the mucous cells on the skin surface of fish, affecting their respiratory function. Additionally, elevated pH values can lead to damage to the gill tissues of fish, influencing the organism’s ion regulation. Alkaline stress further disrupts the ion permeation balance within fish, affecting osmoregulation [5]. In such harsh environmental conditions, the majority of aquatic organisms struggle to survive. In recent years, the continuous expansion of global saline–alkaline areas has been exacerbated by increased water evaporation and rising sea levels due to global warming, further limiting the space for freshwater aquaculture [6]. Therefore, investigating the adaptive growth and metabolic strategies of fish under saline–alkaline stress is crucial for providing theoretical support to address the challenges of fish farming in these environments. Our research holds significant implications for building an ecologically harmonious and sustainably developed fishery industry [7].

The exposure of fish to high concentrations of saline–alkaline environments results in a cascade of physiological issues. These include respiratory metabolism disorders [8], oxidative stress damage to tissues and organs [9], hindered ammonia excretion [10], and imbalances in antioxidant and immune systems [11]. These problems significantly affect normal fish growth, development, and reproduction [12]. Notably, ammonia excretion plays a crucial role in fish adapting to saline–alkaline environments [5]. Under normal conditions, fish eliminate excess nitrogen waste by capturing NH_3_ through H^+^ diffusion. However, in highly saline–alkaline environments, the reduction in H^+^ used for capturing NH_3_ to synthesize NH_4_^+^ in gill cells limits the pathway for NH_3_ excretion. Consequently, NH_3_ accumulates in fish plasma and tissues, leading to ammonia toxicity and potential death [13]. Therefore, maintaining physiological homeostasis relies on timely and effective ammonia excretion after fish ingestion [14]. Under high-pH and high-alkalinity conditions, converting accumulated endogenous ammonia into less toxic compounds, such as glutamine, free amino acids, or urea, becomes the primary pathway for ammonia excretion in fish [15]. Research indicates that under highly saline–alkaline environmental stress, crucian carp (*Carassius auratus*) experience a significant increase in blood ammonia concentration, coupled with hindered ammonia excretion. To counter this challenge, crucian carp activate the glutamate metabolism pathway to increase the synthesis of glutamine and urea, mitigating the harmful effects of ammonia toxicity [16]. In the case of acute carbonate alkaline stress, Nile tilapia (*Oreochromis niloticus*) reach a peak blood ammonia concentration after 12 h and regulate it through the glutamate metabolism pathway [17]. Understanding the regulatory mechanism of the glutamate metabolism pathway on ammonia excretion in fish under saline–alkaline stress at the cellular metabolism level is crucial for further enhancing the adaptation of fish to saline–alkaline environmental stress [16]. However, the significant impact of blocking the glutamate metabolism pathway on ammonia excretion in freshwater teleosts has not been publicly reported. Investigating this issue is of crucial scientific significance for understanding the physiological and metabolic impacts on freshwater teleosts.

Metabolomics, functioning as a tool to intricately link metabolic products with endogenous or exogenous stressors, offers a novel avenue for exploring potential toxic pathways and the adverse effects of environmental exposure on aquatic organisms [18]. Notably, metabolic products serve as the ultimate manifestations of gene expression and enzymatic activities, providing a sensitive and authentic reflection of the overall functional state of organisms in response to environmental stimuli. Currently, this approach is widely applied to unravel the intricate metabolic mechanisms through which aquatic organisms respond to fluctuations in external environmental factors [19,20]. Employing LC-MS/MS-based metabolomics, Su et al. [21] explored the metabolic responses of Mozambique tilapia (*Oreochromis mossambicus*) under varying saline–alkaline concentrations. Their study elucidated its molecular regulatory mechanisms in response to different osmotic pressures. Similarly, utilizing metabolomics techniques, Zhang et al. [22] investigated *Eriocheir sinensis* and revealed its adaptive strategies to low saline–alkaline stress through the activation of ABC transport proteins and purine metabolism. In contrast, the crabs countered a high amount of saline–alkaline stress through pyrimidine metabolism and β-alanine metabolism. Hence, metabolomics has progressively emerged as a leading approach for studying the environmental adaptability of aquatic organisms.

The annual production of crucian carp, a prominent freshwater aquaculture species in China, exceeds 2.76 × 10^6^ million tons, with an aquaculture scale that is steadily increasing each year [23]. Its wide-ranging diet, rapid growth, robust stress resistance, and powerful immune functions have positioned the crucian carp as a reliable model for investigating the physiological mechanisms underlying salinity–alkalinity tolerance in freshwater fish [24]. Previous studies have successfully delved into the toxic effects of environmental stressors, such as microplastics [25], high temperatures [26], and bacterial infections [27], on crucian carp, yielding satisfactory research outcomes. This study aims to utilize UPLC-Q-TOF/MS metabolomics technology to explore the impact of blocking the glutamate metabolism pathway on the serum metabolites of crucian carp under carbonate alkaline stress. At the cellular metabolism level, we seek to unravel the mechanism by which this metabolic pathway influences ammonia excretion in crucian carp. Our overarching goal is to provide new solutions to address the challenge of hindered ammonia excretion faced by freshwater teleosts in saline–alkaline environments.

## 2. Materials and Methods

### 2.1. Instruments and Reagents

Instrumentation and reagents employed in this study include the Triple TOF 5600^+^ mass spectrometer (SCIEX, Framingham, MA, USA), the ACQUITY UPLC ultra-high-performance liquid chromatography system (Waters, Milford, MA, USA), the Allegra X-30R high-speed centrifuge (Beckman Coulter, Pasadena, CA, USA), a vortex mixer (IKA, Staufen, Germany), a Milli-Q A10 ultrapure water system (Millipore, Boston, MA, USA), a programmable ultrasonic processor (Kunshan Ultrasonic Instruments Company, Suzhou, China), and the following specific chemicals: methionine sulfoximine (MSO, Sigma-Aldrich, St. Louis, MI, USA), tricaine methanesulfonate (MS-222, Sigma-Aldrich, St. Louis, MI, USA), sodium bicarbonate, sodium chloride (analytical grade, Tianjin Tiantai Chemical Factory, Tianjin, China), methanol, acetonitrile, formic acid (mass spectrometry grade, Merck, Darmstadt, Germany), physiological saline (Sichuan Kelun Pharmaceutical Company, Chengdu, China), and organic-phase filter membranes (13 mm, 0.22 μm; Anpu, Shanghai, China).

### 2.2. Experimental Design and Feeding Management

In this investigation, we procured healthy crucian carp devoid of any apparent diseases from the Hulan Experimental Station at the Heilongjiang Fisheries Research Institute, Chinese Academy of Fishery Sciences. A total of 540 specimens, with an average weight of 130.67 ± 7.19 g and a body length of 17.83 ± 1.28 cm, were randomly selected and subjected to a two-week acclimation period within indoor recirculating tanks measuring 100 cm × 45 cm × 50 cm. Subsequently, the individuals were randomly allocated to six treatment groups, each with three replicates consisting of 30 fish per replicate. After 30 days of experiments, sodium chloride solution (concentration of 0.9%) and MSO were injected, and MSO solid powder was dissolved in sodium chloride solution to form a 0.056 mol/L solution before injection. Feeding was suspended post-injection, and samples were collected 96 h later. Specifically, the experimental groups comprised the following: C (abdominal cavity injection of 0.9% NaCl), MC (abdominal cavity injection of MSO at a dose of 10 mg/kg fish) [28], L (exposed to 20 mmol/L NaHCO_3_ and subjected to abdominal cavity injection of 0.9% NaCl), ML (exposed to 20 mmol/L NaHCO_3_ and subjected to abdominal cavity injection of MSO at a dose of 10 mg/kg fish), H (exposed to 40 mmol/L NaHCO_3_ and subjected to abdominal cavity injection of 0.9% NaCl), and MH (exposed to 40 mmol/L NaHCO_3_ and subjected to abdominal cavity injection of MSO at a dose of 10 mg/kg fish). The NaHCO_3_ exposure groups were gradually elevated to the experimental concentration at a daily rate of 5 mmol/L. All animal procedures in this study adhered to the Guidelines for Care and Use of Laboratory Animals of Heilongjiang River Fisheries Research Institute of the Chinese Academy of Fishery Sciences and received approval from the Animal Ethics Committee of Heilongjiang River Fisheries Research Institute of Chinese Academy of Fishery Sciences (No. 20210316-002).

During the experimental period, the daily feeding rate was approximately 3% of the fish body weight, with feeding conducted twice a day at 8:30 and 17:30. After each feeding session lasting 30 min, residual feed and feces were promptly removed to prevent water quality contamination. The water temperature was maintained at around (24 ± 1.0) °C. In the control group, the pH was 6.95, while the pH in the 20 mmol/L NaHCO_3_ exposure group was 8.7, and in the 40 mmol/L NaHCO_3_ exposure group, the pH was 9.18. Ammonium nitrogen levels were kept below 1.0 mg/L, and dissolved oxygen levels were maintained above 7.5 mg/L. A partial water change of one-third of the total volume was performed every 3 days, and NaHCO_3_ was supplemented as needed to sustain the experimental concentration (as determined by acid–base titration method for NaHCO_3_ concentration). Five tails were randomly selected from each parallel group (15 tails from each treatment group for a total of 90 tails). After anesthesia with 100 mg/L MS-222, blood was collected from the caudal vein using a sterile 5 mL syringe, transferred to a centrifuge tube, and allowed to stand at 4 °C for 2 h. After centrifugation at 3500 rpm for 10 min, the supernatant was collected and stored at −80 °C for further analysis.

### 2.3. UPLC-Q-TOF/MS Analysis

#### 2.3.1. Sample Preparation

Fifteen serum samples were randomly selected from each treatment group and thawed at 4 °C. A volume of 100 μL was aspirated into 2 mL centrifuge tubes using a pipette. Subsequently, 400 μL of a methanol solution (methanol:water = 4:1, *v*/*v*) [29] was added, followed by vortex mixing. The mixture underwent 20 min of ice bath ultrasonication, with 1 h of settling at −20 °C and centrifugation at 4 °C and 13,000 r/min for 10 min. The resulting 400 μL of supernatant was filtered through a 0.22 μm organic-phase filter membrane into an auto-sampler vial for analysis. Simultaneously, an equal volume of serum samples from each group was mixed to prepare quality control samples (QC) for assessing the stability of the high-resolution mass spectrometer and the reliability of the data.

#### 2.3.2. Detection Using UPLC-Q-TOF/MS

Chromatographic separation was achieved using a BEH C18 column (100 mm × 2.1 mm, 1.7 μm) with a column temperature set to 40 °C. For ESI^+^ ion mode, mobile phase A consisted of a 0.1% formic acid aqueous solution (formic acid:water = 1:1000, *v*/*v*), while mobile phase B comprised a 0.1% formic acid acetonitrile solution (formic acid:acetonitrile = 1:1000, *v*/*v*). In ESI^–^ ion mode, mobile phase A was water, and mobile phase B was acetonitrile. The elution gradient was as follows: 0–2 min, 5–30% B; 2–3 min, 30–70% B; 3–11 min, 70–90% B; 11–12 min, 90–100% B; 12–13 min, 100% B; 13–13.1 min, 100–5% B; and 13.1–15 min, 5% B. The flow rate was set at 0.30 mL/min, and the injection volume was 10 μL.

Mass spectrometric detection was conducted using an electrospray ionization source (ESI) scanning in both positive and negative ion modes. The ion source temperature was set to 550 °C, with ion source voltages of 5500 V and −4500 V for positive and negative ion modes, respectively. The curtain gas (CUR) was maintained at 241.3 kPa and the spraying gas (GAS) at 344.8 kPa. The declustering voltage (DP) was set to 80 V for positive mode and −80 V for negative mode, with collision energies of 35 eV and −35 eV, respectively. TOF MS scan mode was employed for the acquisition of the first-stage mass spectrometry ion scan in the range of M/Z 100–1500 Da. Additionally, information-dependent acquisition (IDA) mode was utilized for the acquisition of the second-stage mass spectrometry ion scan in the range of M/Z 50–1200 Da.

### 2.4. Data Processing

The raw data obtained from UPLC-Q-TOF/MS (.wiff files) were imported into Progenesis QI 2.1 software (Waters Corporation, Milford, MA, USA) for data preprocessing, including peak extraction, alignment, and filtering. This process generated a data matrix containing retention time, mass-to-charge ratio, and peak area. The data matrix was then imported into SIMCA 14.1 software (Umetrics, Umea, Sweden) for pattern recognition. Principal component analysis (PCA) and orthogonal partial least squares discriminant analysis (OPLS-DA) were employed for further analysis to discern metabolic changes among different groups. Metabolites showing significant differences were selected based on having variable importance in projection (VIP) values greater than 1 and a significance level (*p*) less than 0.05, identifying them as differential metabolites (DMs). Further annotation and identification of DMs were performed based on retention time, relative molecular mass, secondary fragment ions, and searches against the Human Metabolome Database (HMDB, http://www.HMDB.ca/ accessed on 6 September 2023) and the Kyoto Encyclopedia of Genes and Genomes (KEGG). The identified DMs were then imported into MetaboAnalyst 5.0 (https://www.metaboanalyst.ca/ accessed on 6 September 2023) for pathway analysis, providing insights into the underlying metabolic pathways associated with the observed changes.

## 3. Results

### 3.1. Principal Component Analysis (PCA) and Orthogonal Partial Least Squares Discriminant Analysis (OPLS-DA) 

**Figure 1:**
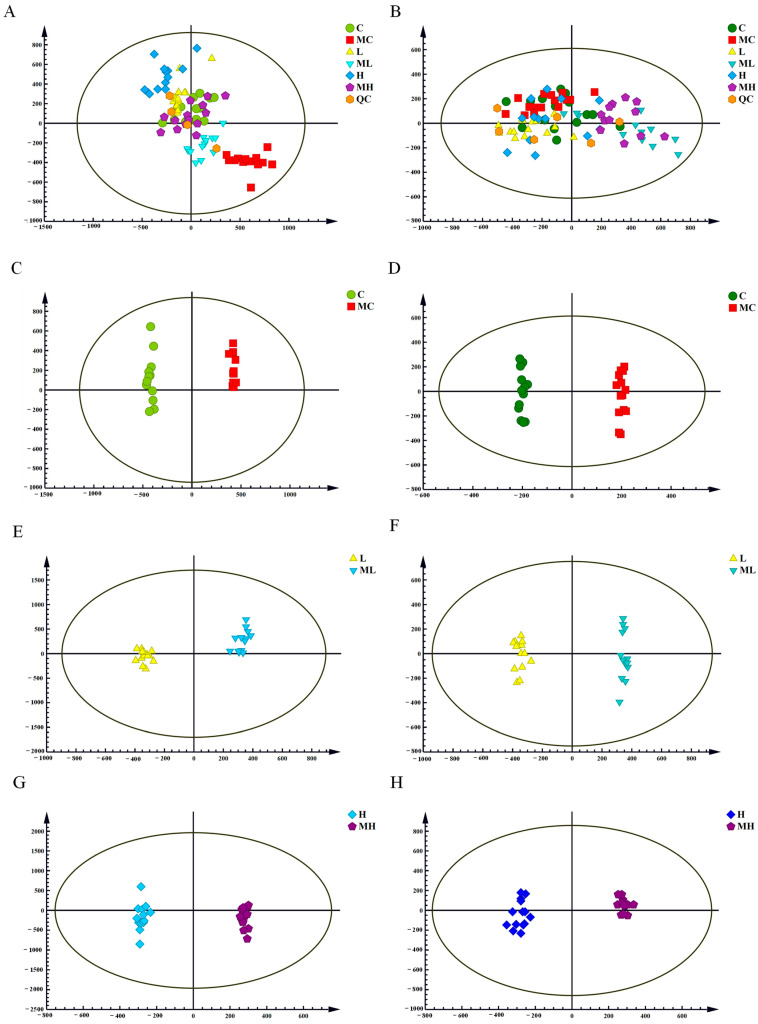
Serum metabolomics PCA and OPLS-DA. Plot of PCA scores in positive and negative ion modes (**A**,**B**); OPLS-DA scores of C vs. MC in positive and negative ion modes (**C**,**D**); OPLS-DA scores of L vs. ML in positive and negative ion modes (**E**,**F**); and validation of OPLS-DA model substitution of H vs. MH in positive and negative ion modes (**G**,**H**); C is the fresh water and injected with NaCl group, M is the MSO injection group, L is the 20 mmol/L NaHCO_3_ stress group, ML is the 20 mmol/L NaHCO_3_ under stress and injected with MSO group, H is the 40 mmol/L NaHCO_3_ stress group, and MH is the 40 mmol/L NaHCO_3_ under stress and injected with MSO group.

The UPLC-Q-TOF/MS data’s PCA score plots in both the positive and negative ion modes are depicted in Figure 1A,B. QC samples cluster tightly in a specific region, indicating the instrument’s high stability and reproducibility, as well as the stability of the samples. However, in the unsupervised principal component analysis model, metabolites could not be clearly differentiated between the experimental and control groups, possibly due to limitations in the PCA model [30]. OPLS-DA models were subsequently established separately in the positive and negative ion modes to further distinguish differences among groups. The models’ fitting capabilities were assessed through Q^2^ (cum) and R^2^Y (cum). Significant separation between groups was observed in the positive and negative ion modes for C vs. MC (Figure 1C,D), L vs. ML (Figure 1E,F), and H vs. MH (Figure 1G,H), indicating substantial disruption in the normal expression of serum metabolites due to the inhibition of the glutamine metabolism pathway. The R^2^Y and Q^2^ values for the positive ion model were 0.968 and 0.912, and for the negative ion model, they were 0.984 and 0.864, respectively. These results suggest that the OPLS-DA models fit well, exhibit a high level of predictability, and are suitable for subsequent data analyses.

### 3.2. Identification of Differentially Expressed Metabolites

**Figure 2:**
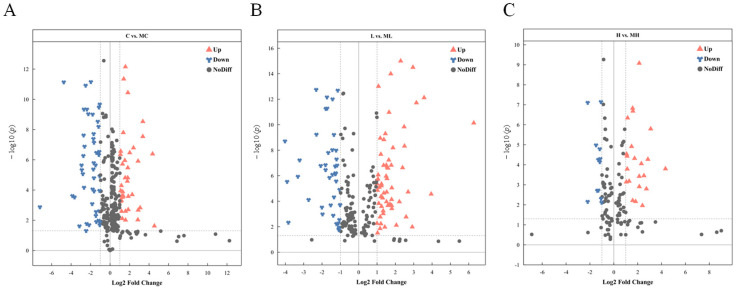
Volcano plots of DMs’ distribution in crucian carp serum after blockade of the glutamate pathway at three different base concentrations (**A**) C vs. MC; (**B**) L vs. ML; and (**C**) H vs. MH.

**Figure 3:**
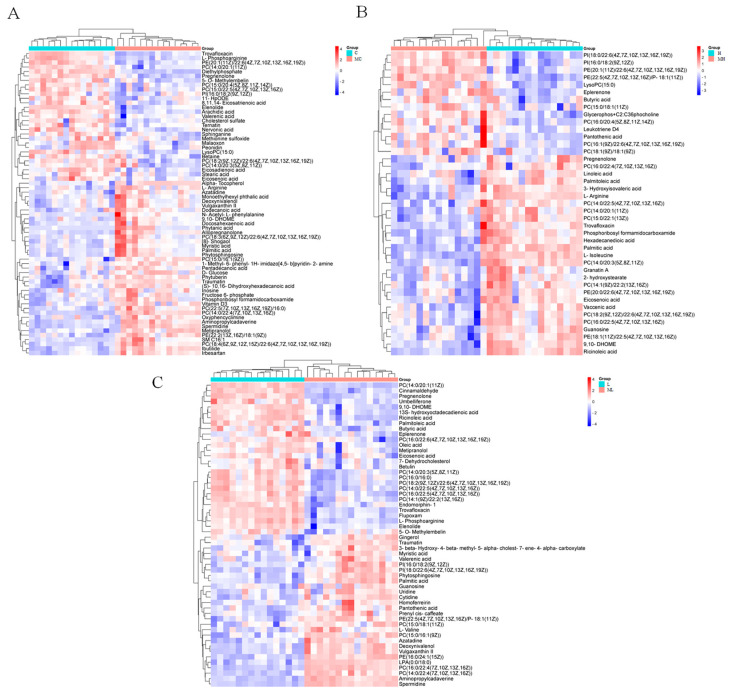
Hierarchical clustering of differential metabolites based on *p* < 0.05 and VIP > 1.0; C is the fresh water and injected with NaCl group; MC is the MSO injection group; L is the 20 mmol/L NaHCO_3_ stress group; ML is the 20 mmol/L NaHCO_3_ under stress and injected with MSO group; H is the 40 mmol/L NaHCO_3_ stress group; MH is the 40 mmol/L NaHCO_3_ under stress and injected with MSO group. (**A**) C vs. MC; (**B**) H vs. MH; and (**C**) L vs. ML.

**Figure 4:**
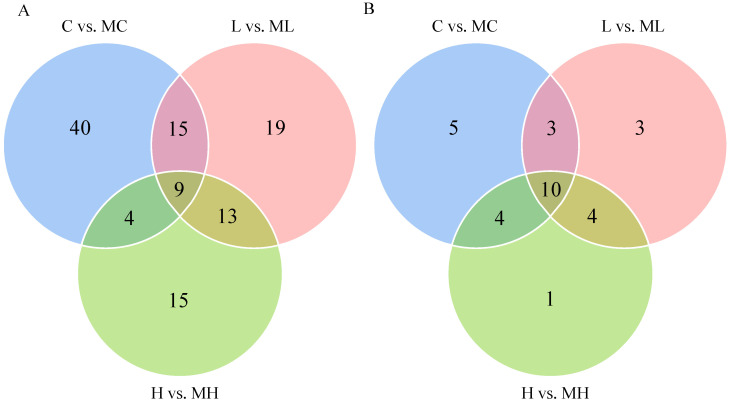
Venn diagram of differential metabolites and metabolic pathways. (**A**) is a Venn diagram of the number of metabolites enriched in comparisons of C vs. MC, L vs. ML, and H vs. MH and their overlaps; (**B**) is a Venn diagram of the number of metabolic pathways enriched in comparisons of C vs. MC, L vs. ML, and H vs. MH and their overlaps.

DMs were identified using VIP > 1 and *p* < 0.05 as criteria for screening. In the comparison between the C and MC groups, 96 DMs were detected (67 increased and 29 decreased), while in the L vs. ML group, 71 DMs were identified (51 increased and 20 decreased). In the H vs. HM group, 61 DMs were characterized (42 increased and 19 decreased). The volcano plot illustrates the distribution of DMs following the disruption of the glutamine metabolism pathway under various NaHCO_3_ stress concentrations (Figure 2). Hierarchical clustering reveals the variation in DMs across the three distinct treatment groups (Figure 3), while the Venn diagram depicts the overlapping differential metabolites between the C and MC, L and ML, and H and HM groups (Figure 4A), as well as the associated metabolic pathways (Figure 4B).

### 3.3. Enrichment Analysis of Metabolic Pathways

**Figure 5:**
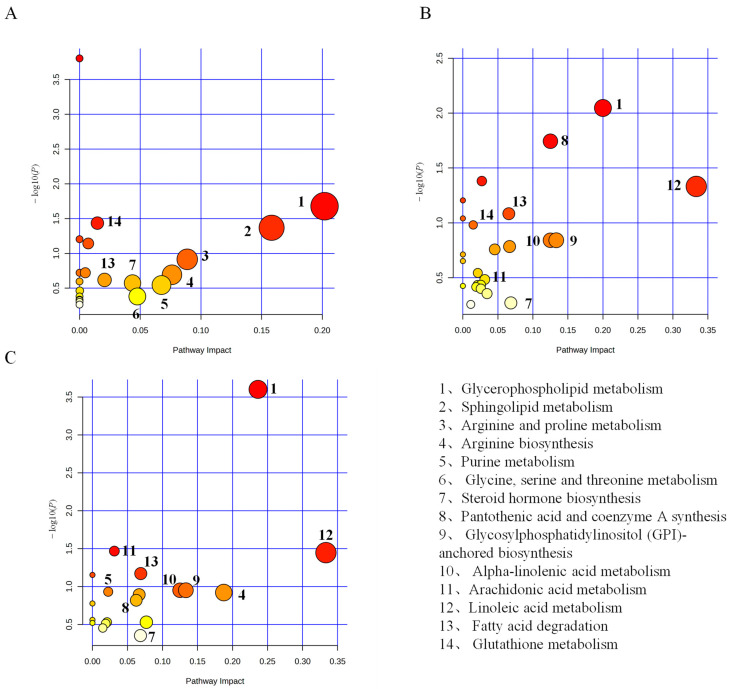
Metabolic pathway enrichment analysis of serum differential metabolites in crucian carp after glutamate pathway blockade. (**A**) C vs. MC; (**B**) L vs. ML; and (**C**) H vs. MH.

**Figure 6:**
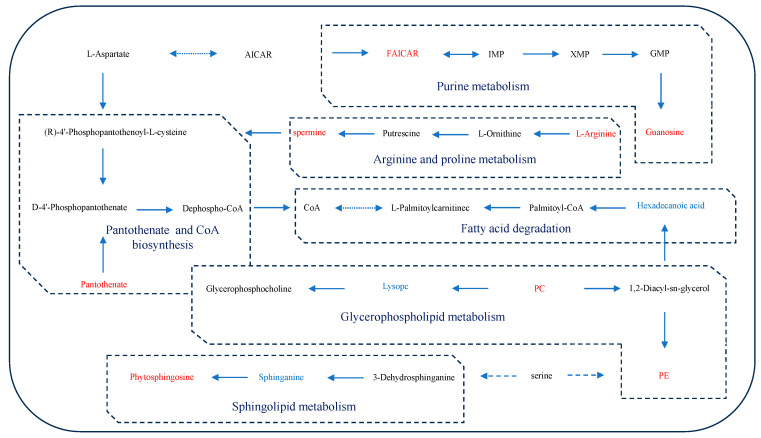
Network diagram of serum differential metabolite–metabolic pathway interactions, with metabolites in red (increased content) or blue (decreased content).

The identified DMs were imported into MetaboAnalyst 5.0 to investigate potential metabolic pathways affected in crucian carp serum under saline–alkaline stress following the disruption of the glutamine metabolism pathway. Our analysis aimed to elucidate changes in pathways related to the inhibition of the glutamine metabolism pathway. In the C vs. MC group, the enriched DMs in crucian carp serum were primarily associated with glycerophospholipid metabolism, sphingolipid metabolism, arginine and proline metabolism, arginine biosynthesis, purine metabolism, glycine, serine, and threonine metabolism, fatty acid degradation, glutathione metabolism, and steroid biosynthesis (Figure 5A). In the L vs. ML group, the DMs were predominantly enriched in linoleic acid metabolism, glycerophospholipid metabolism, pantothenate and CoA biosynthesis, alpha-linolenic acid metabolism, arachidonic acid metabolism, glutathione metabolism, and steroid biosynthesis (Figure 5B). In the H vs. MH group, the enriched DMs were mainly associated with glycerophospholipid metabolism, linoleic acid metabolism, arachidonic acid metabolism, arginine biosynthesis, purine metabolism, arginine and proline metabolism, alpha-linolenic acid metabolism, pantothenate and CoA biosynthesis, and fatty acid degradation (Figure 5C). A further analysis revealed significant impacts on glycerophospholipid metabolism, arachidonic acid metabolism, sphingolipid metabolism, CoA biosynthesis, arginine and proline metabolism, fatty acid degradation, and purine metabolism following the disruption of the glutamine metabolism pathway. Figure 6 illustrates the changes in key metabolites and metabolic pathways. In summary, these results underscore that the inhibition of the glutamine metabolism pathway significantly influences the metabolic processes in crucian carp serum.

## 4. Discussions

Currently, there exists a substantial expanse of barren saline–alkaline land and secondary saline–alkaline water bodies worldwide. Not only do these affected areas increase annually due to salinization, but the utilization efficiency of these resources remains extremely low [31]. Hypercarbonate saline water has high levels of alkalinity (HCO_3_^−^ and CO_3_^2−^) and pH, a complex ionic composition, and other physicochemical characteristics, which will not only lead to fish respiratory and metabolic alkalosis, but will also cause a series of physiological problems, such as ammonia excretion obstruction. Ammonia toxicity has been proven to be the main reason why fish cannot adapt to high-saline and -alkaline environments [16]. Existing studies indicate that the glutamine metabolism pathway can convert endogenous ammonia into the less toxic glutamine–glutamate, thereby preventing ammonia toxicity. However, the mechanistic impact of the glutamine metabolism pathway on the metabolism of freshwater teleosts in saline–alkaline habitats remains unclear [16]. UPLC-Q-TOF/MS metabolomics technology was employed to disrupt the normal functioning of the crucian carp glutamine metabolism pathway through the intraperitoneal injection of MSO in this study. The results revealed that blocking the glutamine metabolism pathway induced oxidative stress and inflammatory responses in crucian carp, leading to disturbances in their lipid metabolism and amino acid synthesis. Furthermore, significant impacts on their energy metabolism and ammonia excretion were observed following the disruption of the glutamine metabolism pathway.

### 4.1. Oxidative Stress

Under normal physiological conditions, intracellular reactive oxygen species (ROS) are maintained in a dynamic equilibrium, with the body’s antioxidant system continuously generating and clearing them [32]. Prolonged environmental stress leads to sustained ROS production, which attack cell membrane lipids and cause lipid peroxidation. This damages the permeability of cell membranes, affecting their normal physiological functions and inducing oxidative stress [33]. Glycerophospholipid metabolism plays an important role in biology and is involved in the maintenance of cell structure, organelle function, and energy homeostasis [34]. Our study found that glycerophospholipid metabolism after the blockade of the glutamate pathway was significantly enriched in treatment groups with different saline concentrations, with significantly increased in phosphatidylcholine (PC) analogs and phosphatidylethanolamine (PE) analogs. PC and PE are major phospholipid components of biological membranes, contributing to the fluidity and integrity of cell membranes, which are vital for maintaining normal physiological functions [35]. Studies on *Scophthalmus maximus* under high-temperature stress at 24 °C have shown that glycerophospholipid metabolism is significantly affected, with a decrease in PC content. This suggests that the flatfish regulates genes and metabolites related to lipid metabolism to maintain membrane balance, inhibit lipid deposition, and alleviate meat deterioration caused by high-temperature stress [36]. Additionally, research on *Scatophagus argus* under salt stress found that both high and low amounts of salt stress led to a significant downregulation of genes (*cept1* and *pla2g4a*) associated with glycerophospholipid metabolism. This indicates a reduced efficiency in the conversion of phosphatidylserine, and changes in cell membrane structure and energy storage may occur for the organism to adapt to different salinity conditions [37]. Therefore, we hypothesize that the disruption of the glutamine metabolism pathway leads to more severe damage to crucian carp cell membranes. In response, crucian carp increase the levels of PC and PE compounds to enhance their glycerophospholipid metabolism, maintaining membrane homeostasis and counteracting the cell membrane damage caused by the disruption of the glutamine metabolism pathway.

Arachidonic acid regulates cell membrane fluidity and has an indirect impact on cell signaling pathways, including the mediation of inflammatory responses and synaptic transmission. Moreover, it is closely linked to oxidative stress, which can arise from the production of substantial amounts of reactive oxygen species [38]. Typically, oxidative stress causes inflammation to occur [39]. Oxidative stress is a state in which there is an imbalance between oxidation and antioxidant effects in the body, which tends to oxidize, leading to the inflammatory infiltration of neutrophils and increased secretion of proteolytic enzymes. Additionally, the production of a large number of oxidative intermediates oxidizes or damages DNA, proteins, and lipids directly or indirectly and can lead to physiological and pathological responses in cells and tissues [40]. At the same time, the body in the external environment under the stimulation of macrophages can generate a large number of inflammatory substances; these high concentrations of inflammatory substances are also an important factor in inducing the body to produce an inflammatory response [41]. Recent research by Zhao et al. [42] demonstrated significant changes in the expression levels of genes involved in arachidonic acid metabolism in the gills and kidneys of *Leuciscus waleckii* under highly alkaline conditions, leading to the generation of an immune response. Similarly, under carbonate saline–alkaline stress, the disruption of arachidonic acid metabolism in the liver of crucian carp resulted in a significant increase in the concentration of metabolites closely related to arachidonic acid metabolism, leading to inflammation in the crucian carp’s liver cells [43]. Our study found that metabolites related to immune and inflammatory responses, such as arachidonic acid and leukotriene A4 (LTA4), were significantly enriched in the MH group. Hence, we infer that the disruption of the glutamine metabolism pathway induces inflammation in crucian carp, further triggering an immune response.

Glutathione manifests in two forms within the cell-oxidized glutathione (GSSG) and reduced glutathione (GSH), with GSH being synthesized from GSSG through the enzymatic action of glutathione reductase. Serving as a crucial cellular antioxidant, GSH effectively neutralizes free radicals and other harmful oxidants, thereby safeguarding cells from oxidative damage [44]. Peroxides and free radicals, which are the products of oxidative stress, can have toxic effects on cells. Fish can respond to oxidative stress caused by external environmental factors through the glutathione metabolic pathway [45]. Under heat stress conditions, *Oncorhynchus mykiss* activated the glutamate–glutamine metabolic pathway to respond to oxidative damage caused by heat stress [46]. There is a relationship between spermidine and glutathione metabolism. Spermidine plays an important role in glutathione metabolism; in particular, under oxidative stress conditions, spermidine can inhibit glutathione enzyme activity, which reduces glutathione catabolism and protects cells from oxidative stress and inflammatory damage [47]. Wu et al. investigated sex-specific metabolites in the corpora cavernosa of *Holothuria scabra* using an LC-MS/MS metabolomics approach. They discovered that male corpora cavernosa contained metabolites such as spermidine, which were involved in glutathione metabolism and spermidine proline metabolism [48]. Our results revealed that the glutathione metabolic pathway was significantly enriched in both the MC and ML groups after the blockade of the glutamate pathway, in which the spermidine involved in glutathione metabolism had a high concentration. Thus, we hypothesize that we could improve the antioxidant capacity of the organism by activating the glutathione metabolic pathway. In addition, we could inhibit the catabolism of glutathione enzyme and reduce the oxidative stress damage in crucian carp by increasing the concentration of spermidine.

Apoptosis, responsible for tissue remodeling, is a common programmed cell death regulated by genes to maintain cellular homeostasis [5]. Sphingolipids ensure the structural integrity of the cell membrane and modulate processes such as apoptosis, proliferation, and senescence. Specifically, sphinganine acts as a stimulant, enhancing the permeability of lysosomal membranes [49]. Our study found that a significant enrichment of sphingolipid metabolism was observed in the glutamate metabolism pathway blockade experimental group compared to the control group. Conversely, the content of sphinganine, a metabolite closely associated with sphingolipid metabolism, exhibited a marked decrease. This suggests that the disruption of glutamate metabolism pathways leads to the dysregulation of the carp’s sphingolipid metabolism, subsequently inducing apoptosis and adversely affecting its cellular well-being. This aligns closely with the findings of Ding et al. [50], who reported similar disruptions in sphingolipid metabolism and apoptosis induction in crucian carp under saline–alkaline stress.

### 4.2. Obstruction of Ammonia Excretion

The arginine and proline pathways link arginine, proline, glutamate, and their intermediates [51], while urea is formed primarily by the catabolism of uric acid or arginine [52]. Gao et al. investigated the physiological changes in gill tissues of *Coilianasus* under salt stress and found that the proline metabolic pathway was activated in order to resist salt stress as a way to improve its low salt tolerance [53]. Dong et al. characterized the physiological and metabolic changes in *Macrobrachiumrosenbergii* under ammonia stress and showed that high levels of environmental ammonia stress increased the body’s glutamate and arginine levels, and these two amino acids may be involved in the urea cycle to synthesize glutamine or urea to eliminate ammonia toxicity [54]. Our study found that the blockade of the glutamate metabolism pathway resulted in a significant increase in the L-arginine content, suggesting that the blockade of the glutamate metabolism pathway may interfere with amino acid metabolism in crucian carp. The elevation of L-arginine indicates that the blockage of glutamate metabolic pathway leads to the further serious obstruction of ammonia excretion in crucian carp, which prevents ammonia from being converted into urea for normal excretion from the body, thus leading to the accumulation of L-arginine. It has been shown that L-arginine can be cleaved to ornithine and urea catalyzed by arginase (ARG), as well as catalyzed by intracellular nitric oxide synthase (NOS), which undergoes a multistep redox reaction with oxygen molecules and continuously generates endogenous NO molecules in low concentrations [55]. Simultaneously, NO reacts easily with oxygen radicals, producing the more harmful oxidant, peroxynitrite anion (ONOO^−^), which then causes the cytotoxic reaction. Thus, high concentrations of NO amplify the damaging effects of oxygen radicals and the inflammatory response, as well as the extent of tissue damage [56]. Our study found a significant increase in the L-arginine concentration after the blockade of the glutamate pathway. We hypothesize that high concentrations of L-arginine might be converted to high concentrations of NO, catalyzed by NOS, thereby exacerbating oxidative damage and inflammatory responses within the cell.

Purine metabolism constitutes a vital pathway for the degradation of nitrogenous compounds, serving as an effective route for the excretion of uric acid or urea to reduce the accumulation of nitrogen waste in aquatic organisms [57]. In this process, guanine and adenine can be converted into xanthine, which further transforms into NH_3_ and CO_2_ through intermediate products such as uric acid, allantoin, allantoic acid, and urea, eventually being expelled from the body [58]. Current research indicates that under ammonia stress, the expression of most genes associated with the purine metabolism pathway in *L. vannamei*, including the *XDH* gene, a key enzyme in purine metabolism, is significantly downregulated. The reduction in purine metabolism is considered a self-protective mechanism to prevent excessive uric acid synthesis, which could impose an undue physiological burden [59]. The purine metabolism of *Coilianasus* was disturbed under ammonia stress, and the expression of genes related to purine metabolism was significantly suppressed, which reduced the effects of ammonia stress by decreasing the purine metabolism, even though the inflammatory response caused by ammonia stress still existed up to 24 h after the freshwater recovered [60]. In a metabolomics and transcriptomics study of Nile tilapia, Zhang found that dietary LBP activated purine metabolism and increased nitrogenous waste excretion in tilapia, thereby promoting tilapia growth [61]. We found that guanosine, which is involved in purine metabolism, exhibited high concentrations after the blockade of the glutamate pathway and the purine metabolism was significantly activated, suggesting that ammonia excretion in crucian carp was blocked after the glutamate pathway was blocked. Moreover, it was possible to alleviate the phenomenon of ammonia toxicity by activating the purine metabolism to increase nitrogen-containing waste excretion in the body.

### 4.3. Energy Metabolism Disorder

Pantothenic acid, also known as vitamin B5, serves as a crucial precursor in the biosynthesis of coenzyme A (CoA), and organisms can promote lipid and carbohydrate metabolism by upregulating pantothenic acid to enhance pantothenic acid and CoA biosynthesis [62]. CoA is a vital cofactor in cellular growth, participating in numerous metabolic reactions, including the synthesis of phospholipids, fatty acid synthesis and degradation, and the tricarboxylic acid (TCA) cycle [63]. The TCA cycle is the primary pathway for ATP production in the body, providing over 90% of the energy required for an organism’s life processes [64]. A study by Zhang et al. [65] on the effects of the water flow rate stress on the liver of *Trachinotus ovatus* through metabolomics revealed that, compared to the static water group, both the medium-flow and high-flow groups showed an increase in the pantothenic acid content. This suggested that flow rate stress promotes the TCA cycle in the liver of ovate pomfret, providing energy for their movement through enhanced aerobic respiration. Our study found that a significant increase in the pantothenic acid content was observed after the blockade of the glutamate metabolism pathway, indicating an enhancement in the synthesis of pantothenic acid and CoA. Consequently, we hypothesize that carp experience an energy deficiency following the blockade of the glutamate metabolism pathway. Crucian carp respond by augmenting the synthesis of pantothenic acid and CoA, further promoting the metabolism of lipids and carbohydrates to enhance energy metabolism and counteract the adverse effects of the blockade of the glutamate metabolism pathway. This aligns with the findings of a study on *Anguilla anguilla*, in which the upregulation of pantothenic acid and CoA metabolism was observed to promote energy production [66].

Environmental stress can accelerate fat breakdown, providing free fatty acids as a primary source of energy for fish metabolism [67]. Studies have shown that fatty acid anabolism and catabolism, including fatty acid elongation, fatty acid degradation, unsaturated fatty acid biosynthesis, and fatty acid β-oxidation, are activated in several tissues, including intestine, hepatopancreas, and muscle, in *Gymnocypris przewalskii* from Qinghai Lake under cold stress, suggesting that the naked carp dependently utilize fatty acids as an energy primary source [68]. Another study demonstrated that under crowding stress, immune indicators in hybrid sturgeon (♀*Acipenser baerii* × ♂*Acipenser schrenckii*) did not show significant changes. However, pathways related to fatty acid degradation, gluconeogenesis, and glycogen breakdown were significantly activated to account for the increased energy expenditure in sturgeon under crowding stress [69]. Our study found that after the blockade of the glutamate metabolism pathway under carbonate alkaline stress, metabolites closely associated with the fatty acid degradation pathway exhibited a significant decrease. This suggests an abnormality in energy metabolism in carp after the blockade of the glutamate metabolism pathway, indicating that carp maintain energy homeostasis by consuming internal fatty acids.

## 5. Conclusions

This study employed UPLC-Q-TOF/MS untargeted metabolomics technology to investigate the impact of the blockade of the glutamate metabolism pathway on ammonia excretion in crucian carp under saline–alkaline environmental stress. This study revealed that the blockage of the glutamate metabolic pathway disrupted lipid metabolism, including glycerophospholipid metabolism, arachidonic acid metabolism, and sphingolipid metabolism, leading to the development of an inflammatory response in crucian carp. The accumulation of L-arginine may be converted into high concentrations of NO by the catalytic action of NOS, which exacerbates intracellular oxidative damage and inflammation, and may cause the disruption of the cellular membrane structure and function. Simultaneously, purine metabolism, as well as the synthesis pathways of arginine and proline, were significantly enriched, indicating that the blockade of the glutamate metabolism pathway hindered ammonia excretion in crucian carp, resulting in the accumulation of nitrogenous waste in the body. The crucian carp responded by upregulating their purine metabolism to alleviate the ammonia toxicity. In addition, the crucian carp responded to energy abnormalities caused by the blockade of the glutamine pathway by activating pantothenic acid and coenzyme A synthesis as well as fatty acid degradation pathways to maintain energy homeostasis in vivo. At the metabolic level, this study elucidated the adverse effects of the blockade fo the glutamate metabolism pathway on ammonia excretion in crucian carp under saline–alkaline environmental stress. It provided new insights into the challenges of ammonia excretion in fish under saline–alkaline conditions, holding significance for those seeking to effectively utilize saline–alkaline water resources in China.

## Data Availability

Data are available on request.

## References

[1] Yao Z., Lai Q., Zhou K., Rizalita R.E., Wang H. (2010). Developmental biology of medaka fish (*Oryzias latipes*) exposed to alkalinity stress. J. Appl. Ichthyol..

[2] Sherif A.H., Okasha L.A., Kassab A.S., Abass M.E., Kasem E.A. (2024). Long-term exposure to lead nitrate and zinc sulfate Nile tilapia impact the *Aeromonas hydrophila* treatment. Mol. Biol. Rep..

[3] Okomoda V.T., Isah S., Solomon S.G., Ikhwanuddin M. (2024). Salinity tolerance in *Clarias gariepinus* (Burchell, 1822): Insight on blood parameter variations and gill histological changes. Fish Physiol. Biochem..

[4] Linlin A., Zhang Y., Xu B., Zhang H., Li Y., Wang L., Liang J., Zhou W., Feng Z., Zhang H. (2023). Comprehensive analyses of annexins in naked carp (*Gymnocypris przewalskii*) unveil their roles in saline-alkaline stress. Aquaculture.

[5] Tao S., Li X., Wang J., Bai Y., Wang J., Yang Y., Zhao Z. (2023). Examination of the relationship of carbonate alkalinity stress and ammonia metabolism disorder-mediated apoptosis in the Chinese mitten crab, *Eriocheir sinensis*: Potential involvement of the ROS/MAPK signaling pathway. Aquaculture.

[6] Zhou H., Yao T., Zhang T., Sun M., Ning Z., Chen Y., Mu W. (2024). Effects of chronic saline-alkaline stress on gill, liver and intestinal histology, biochemical, and immune indexes in Amur minnow (*Phoxinus lagowskii*). Aquaculture.

[7] Wen J., Chen S.L., Xu W.Y., Zheng G.D., Zou S.M. (2023). Effects of high NaHCO_3_ alkalinity on growth, tissue structure, digestive enzyme activity, and gut microflora of grass carp juvenile. Environ. Sci. Pollut. Res..

[8] Fan F., Zhang Q., Ma Y., Hou M., Sa R., Ma J., Lü X. (2018). Improving biological traits by soda alkali-saline land diking for fish. Trans. Chin. Soc. Agric. Eng..

[9] Fan Z., Wu D., Li J., Li C., Zheng X., Wang L. (2022). Phosphorus Nutrition in Songpu Mirror Carp (*Cyprinus carpio Songpu*) during Chronic Carbonate Alkalinity Stress: Effects on Growth, Intestinal Immunity, Physical Barrier Function, and Intestinal Microflora. Front. Immunol..

[10] Li H., Lai Q., Yao Z., Liu Y., Gao P., Zhou K., Sun Z. (2020). Ammonia excretion and blood gas variation in naked carp (*Gymnocypris przewalskii*) exposed to acute hypoxia and high alkalinity. Fish Physiol. Biochem..

[11] Song L., Zhao Y., Song Y., Zhao L., Ma C., Zhao J. (2021). Effects of saline-alkaline water on growth performance, nutritional processing, and immunity in Nile tilapia (*Oreochromis niloticus*). Aquaculture.

[12] Zhang R., Shi X., Liu Z., Sun J., Sun T., Lei M. (2023). Histological, Physiological and Transcriptomic Analysis Reveal the Acute Alkalinity Stress of the Gill and Hepatopancreas of *Litopenaeus vannamei*. Mar. Biotechnol..

[13] Chang Y.M., Liang L.Q. (2021). Advances of research of physiological and molecular mechanisms related to alkali-saline adaptation for fish species inhabiting alkali-saline water. J. Fish. China.

[14] Zhou C., Xu L., Song H., Feng J., Hu Z., Yang M.J., Shi P., Li Y.R., Guo Y.J., Li H.Z. (2023). Examination of the regulation of energy metabolism, antioxidant response, and ammonia detoxification in hard clam, *Mercenaria mercenaria*, under hypersalinity stress. Aquaculture.

[15] Lv L., Ren J., Zhang H., Sun C., Dong Y., Lin Z. (2022). Transcriptomic analysis of gill and hepatopancreas in razor clam (*Sinonovacula constricta*) exposed to acute ammonia. Front. Mar. Sci..

[16] Liu Y., Yao M., Li S., Wei X., Ding L., Han S., Wang P., Lv B., Chen Z., Sun Y. (2022). Integrated application of multi-omics approach and biochemical assays provides insights into physiological responses to saline-alkaline stress in the gills of crucian carp (*Carassius auratus*). Sci. Total Environ..

[17] Tu H.Q., Zhao J.L., Zhao Y. (2018). Study on the timing sequence of two pathway of *Oreochromis niloticus* ammonia metabolism under the stress of carbonate alkalinity. Freshw. Fish..

[18] Sun Y.C., Wu S., Du N.N., Song Y., Xu W. (2018). High-throughput metabolomics enables metabolite biomarkers and metabolic mechanism discovery of fish in response to alkalinity stress. RSC Adv..

[19] Ding L., Liu Y.J., Wei X.F., Geng C.Y., Liu W.Z., Han L., Yuan F.Y., Wang P., Sun Y.C. (2023). Effects of α-ketoglutarate supplementation on serum metabolism of crucian carp under carbonate alkaline stress based on UPLC-Q-TOF/MS metabolomics. J. Fish. Sci. China.

[20] Li S.W., Han S.C., Liu Y.J., Ding L., Wei X.F., Wang P., Sun Y.C. (2022). Metabolomics of rainbow trout liver under heat stress. J. Fish. Sci. China.

[21] Su H., Li Y., Ma D., Fan J., Zhong Z., Zhu H. (2023). Metabolism responses in the intestine of *Oreochromis mossambicus* exposed to salinity, alkalinity and salt-alkalinity stress using LC-MS/MS-based metabolomics. Comp. Biochem. Physiol. Part D Genom. Proteom..

[22] Zhang R., Zhao Z., Li M., Luo L., Wang S., Guo K., Xu W. (2023). Metabolomics analysis reveals the response mechanism to carbonate alkalinity toxicity in the gills of *Eriocheir sinensis*. Comp. Biochem. Physiol. Part C Toxicol. Pharmacol..

[23] Chen Y., Rong H.N., Liu Z.Y., Chen H.C., Wang Z.Z. (2022). Differential analysis on the effect of biological phenotypes on body weight of *Carassius auratus* gibelio in two pond aquaculture modes. Oceanol. Limnol. Sin..

[24] Choi J.H., Lee J.H., Jo A.H., Choi Y.J., Choi C.Y., Kang J.C., Kim J.H. (2023). Microplastic polyamide toxicity: Neurotoxicity, stress indicators and immune responses in crucian carp, *Carassius carassius*. Ecotoxicol. Environ. Saf..

[25] Kim J.A., Kim M.J., Park Y.S., Kang C.K., Kim J.H., Choi C.Y. (2023). Effects of microfiber and bead microplastic exposure in the goldfish *Carassius auratus*: Bioaccumulation, antioxidant responses, and cell damage. Aquat. Toxicol..

[26] Lacy B., Rahman M.S. (2022). Interactive effects of high temperature and pesticide exposure on oxidative status, apoptosis, and renin expression in kidney of goldfish: Molecular and cellular mechanisms of widespread kidney damage and renin attenuation. J. Appl. Toxicol..

[27] Wang S., Liu S., Wang C., Ye B., Lv L., Ye Q., Xie S., Hu G., Zou J. (2022). Dietary Antimicrobial Peptides Improve Intestinal Function, Microbial Composition and Oxidative Stress Induced by *Aeromonas hydrophila* in Pengze Crucian Carp (*Carassius auratus* var. Pengze). Antioxidants.

[28] Wilkie M.P., Stecyk J.A., Couturier C.S., Sidhu S., Sandvik G.K., Nilsson G.E. (2015). Reversible brain swelling in crucian carp (*Carassius carassius*) and goldfish (*Carassius auratus*) in response to high external ammonia and anoxia. Comp. Biochem. Physiol. Part A Mol. Integr. Physiol..

[29] Liu Y.J., Yao M.Z., Li S.W., Chen Z.X., Wang P., Sun Y.C. (2022). Research on Metabolomics Analysis Method for Fish Gills Target Organs Based on UPLC-QTOFMS. J. Instrum. Anal..

[30] Zhang H., Zhang X., Zhao X., Xu J., Lin C., Jing P., Hu L., Zhao S., Wang X., Li B. (2019). Discrimination of dried sea cucumber (*Apostichopus japonicus*) products from different geographical origins by sequential windowed acquisition of all theoretical fragment ion mass spectra (SWATH-MS)-based proteomic analysis and chemometrics. Food Chem..

[31] Ouyang H., Deng N., Xu J., Huang J., Han C., Liu D., Liu S., Yan B., Han L., Li S. (2023). Effects of hyperosmotic stress on the intestinal microbiota, transcriptome, and immune function of mandarin fish (*Siniperca chuatsi*). Aquaculture.

[32] Yun X., Zhou J., Wang J., Li Q., Wang Y., Zhang W., Fan Z. (2023). Biological toxicity effects of florfenicol on antioxidant, immunity and intestinal flora of zebrafish (*Danio rerio*). Ecotoxicol. Environ. Saf..

[33] Hua M., Deng Q., Qiu M., Deng Y., Sun L., Fang Z., Liao J., Zhao J., Gooneratne R. (2023). Iturin A Strongly Inhibits the Growth and T-2 Toxin Synthesis of Fusarium oxysporum: A Morphological, Cellular, and Transcriptomics Study. Foods.

[34] Zhang J., Zhu Z., Huang J., Yang H., Wang Q., Zhang Y. (2023). Analyzing the impact and mechanism of bisphenol A on testicular lipid metabolism in *Gobiocypris rarus* through integrated lipidomics and transcriptomics. Ecotoxicol. Environ. Saf..

[35] Zhu S., Ye M., Xu J., Guo C., Zheng H., Hu J., Chen J., Wang Y., Xu S., Yan X. (2015). Lipid profile in different parts of edible jellyfish *Rhopilema esculentum*. J. Agric. Food Chem..

[36] Zhao T., Ma A., Yang S., Huang Z. (2021). Integrated metabolome and transcriptome analyses revealing the effects of thermal stress on lipid metabolism in juvenile turbot *Scophthalmus maximus*. J. Therm. Biol..

[37] Chen J., Cai B., Tian C., Jiang D., Shi H., Huang Y., Zhu C., Li G., Deng S. (2023). RNA Sequencing (RNA-Seq) Analysis Reveals Liver Lipid Metabolism Divergent Adaptive Response to Low-and High-Salinity Stress in Spotted Scat (*Scatophagus argus*). Animals.

[38] Li X., Shen Y., Bao Y., Wu Z., Yang B., Jiao L., Zhang C., Tocher D.R., Zhou Q., Jin M. (2022). Physiological responses and adaptive strategies to acute low-salinity environmental stress of the euryhaline marine fish black seabream (*Acanthopagrus schlegelii*). Aquaculture.

[39] Fang M., Lei Z., Ruilin M., Jing W., Leqiang D. (2023). High temperature stress induced oxidative stress, gut inflammation and disordered metabolome and microbiome in tsinling lenok trout. Ecotoxicol. Environ. Saf..

[40] Ferreira I.A., Peixoto D., Losada A.P., Quiroga M.I., do Vale A., Costas B. (2023). Early innate immune responses in European sea bass (*Dicentrarchus labrax L*.) following *Tenacibaculum maritimum* infection. Front. Immunol..

[41] Yao M.X., Yu H.X., Mo H.L., Zhang Z.H., Song Q.C., Liu Q., Yang Q.Y., Wang L.X., Li Y. (2023). Structural and pharmacological characterization of a medium-chain fatty acid receptor GPR84 in common carp (*Cyprinus carpio*). Dev. Comp. Immunol..

[42] Zhao X.F., Liang L.Q., Liew H.J., Chang Y.M., Sun B., Wang S.Y., Mi B.H., Zhang L.M. (2021). Identification and analysis of long non-coding RNAs in *Leuciscus waleckii* adapted to highly alkaline conditions. Front. Physiol..

[43] Wei X.F., Liu Y.J., Li S.W., Ding L., Han S.C., Chen Z.X., Lu H., Wang P., Sun Y.C. (2023). Stress response and tolerance mechanisms of NaHCO_3_ exposure based on biochemical assays and multi-omics approach in the liver of crucian carp (*Carassius auratus*). Ecotoxicol. Environ. Saf..

[44] da Silva D.O., Ratko J., Côrrea A.P.N., da Silva N.G., Pereira D.M.C., Schleger I.C., Neundorf A.K.A., de Souza M.R.D.P., Herrerias T., Donatti L. (2024). Assessing physiological responses and oxidative stress effects in *Rhamdia voulezi* exposed to high temperatures. Fish Physiol. Biochem..

[45] Liu Y., Wang J., Ding J., Zhang Y., Hou C., Shen W., Wu X., Zhu J. (2024). Effects of hypoxia stress on oxidative stress, apoptosis and microorganisms in the intestine of large yellow croaker (*Larimichthys crocea*). Aquaculture.

[46] Li L., Liu Z., Quan J., Lu J., Zhao G., Sun J. (2022). Metabonomics analysis reveals the protective effect of nano-selenium against heat stress of rainbow trout (*Oncorhynchus mykiss*). J. Proteom..

[47] Teng M., Zhao X., Wu F., Wang C., Wang C., White J.C., Zhao W., Zhou L., Yan S., Tian S. (2022). Charge-specific adverse effects of polystyrene nanoplastics on zebrafish (*Danio rerio*) development and behavior. Environ. Int..

[48] Wu F.F., Cheng C.H., Chen T., Zhang X., Wu X.F., Jiang X., Ren C.H., Hu C.Q. (2021). Study on Sex Differential Metabolites and Metabolic Pathway of Parental Tropical Sea Cucumbers *Holothuria scabra*. Prog. Fish. Sci..

[49] Li Q.Q., Xiang Q.Q., Lian L.H., Chen Z.Y., Luo X., Ding C.Z., Chen L.Q. (2021). Metabolic profiling of nanosilver toxicity in the gills of common carp. Ecotoxicol. Environ. Saf..

[50] Ding L., Liu Y., Wei X., Geng C., Liu W., Han L., Yuan F., Wang P., Sun Y. (2023). Effects of Saline-Alkaline Stress on Metabolome, Biochemical Parameters, and Histopathology in the Kidney of Crucian Carp (*Carassius auratus*). Metabolites.

[51] Zhang J.M., Fu B., Li Y.c., Sun J.H., Xie J., Wang G.J., Tian J.J., Kaneko G., Yu E.M. (2023). The effect of nitrite and nitrate treatment on growth performance, nutritional composition and flavor-associated metabolites of grass carp (*Ctenopharyngodon idella*). Aquaculture.

[52] Fauzi I.A., Haga Y., Kondo H., Hirono I., Satoh S. (2020). Dietary citrulline improves survival of rainbow trout *Oncorhynchus mykiss* juveniles challenged with *Vibrio anguillarum*. Aquaculture.

[53] Gao J., Xu G., Xu P. (2021). Gills full-length transcriptomic analysis of osmoregulatory adaptive responses to salinity stress in *Coilia nasus*. Ecotoxicol. Environ. Saf..

[54] Dong X., Liu Q., Kan D., Zhao W., Guo H., Lv L. (2020). Effects of ammonia-N exposure on the growth, metabolizing enzymes, and metabolome of *Macrobrachium rosenbergii*. Ecotoxicol. Environ. Saf..

[55] Yang P., Deng F., Yuan M., Chen M., Zeng L., Ouyang Y., Chen X., Zhao B., Yang Z., Tian Z. (2023). Metabolomics reveals the defense mechanism of histidine supplementation on high-salt exposure-induced hepatic oxidative stress. Life Sci..

[56] Pérez de la Lastra J.M., Juan C.A., Plou F.J., Pérez-Lebeña E. (2022). The Nitration of Proteins, Lipids and DNA by Peroxynitrite Derivatives-Chemistry Involved and Biological Relevance. Stresses.

[57] Duan Y., Nan Y., Zhu X., Yang Y., Xing Y. (2023). The adverse impacts of ammonia stress on the homeostasis of intestinal health in Pacific white shrimp (*Litopenaeus vannamei*). Environ. Pollut..

[58] Chen Y., Wu X., Li P., Liu Y., Song M., Li F., Ou J., Lai J. (2023). Integrated metabolomic and transcriptomic responses to heat stress in a high-altitude fish, *Triplophysa siluroides*. Fish Shellfish. Immunol..

[59] Lei Y., Yuan Z., Zeng Q., Wan B., Liu J., Wang W. (2023). Dynamic N6-methyladenosine RNA methylation landscapes reveal epi-transcriptomic modulation induced by ammonia nitrogen exposure in the Pacific whiteleg shrimp *Litopenaeus vannamei*. J. Hazard. Mater..

[60] Gao J., Zhu Y., Guo Z., Xu G., Xu P. (2020). Transcriptomic analysis reveals different responses to ammonia stress and subsequent recovery between *Coilia nasus* larvae and juveniles. Comp. Biochem. Physiol. Part C Toxicol. Pharmacol..

[61] Zhang X., Xiao J., Guo Z., Zhong H., Luo Y., Wang J., Tang Z., Huang T., Li M., Zhu J. (2022). Transcriptomics integrated with metabolomics reveals the effect of *Lycium barbarum* polysaccharide on apoptosis in Nile tilapia (*Oreochromis niloticus*). Genomics.

[62] Yuan L., Fan L., Dai H., He G., Zheng X., Rao S., Yang Z., Jiao X.A. (2023). Multi-omics reveals the increased biofilm formation of *Salmonella typhimurium* M3 by the induction of tetracycline at sub-inhibitory concentrations. Sci. Total Environ..

[63] Gao Y., Xie Z., Qian J., Tu Z., Yang C., Deng Y., Xue Y., Shang Y., Hu M., Wang Y. (2023). Effects of diel-cycling hypoxia and salinity on lipid metabolism and fatty acid composition of the oyster *Crassostrea hongkongensis*. Mar. Environ. Res..

[64] Hou L., Wang M., Li H., Zhu L., Kong X., Gu W., Bi K., Du J., Meng Q. (2023). Integrated pathological, proteomic and metabolomic analyses reveal significant changes of *Eriocheir sinensis* hepatopancreatic in response to the microsporidian *Hepatospora eriocheir* infection. Aquaculture.

[65] Zhang J., Dai J.Y., Lai X.H., Liu X.X., Zhang H., Wang X.F., Tang B.G. (2023). Metabolomic analysis of *Trachinotus ovatus* under flow velocity stress. Haiyang Xuebao.

[66] Yu Y., Zhao P., Zhai S. (2022). Dietary bile acids supplementation mainly regulates the amino acid metabolic pathways without decreasing bile acids levels in the liver of farmed European eel (*Anguilla anguilla*) juveniles. Aquac. Rep..

[67] Deng W., Sun J., Chang Z.G., Gou N.N., Wu W.Y., Luo X.L., Zhou J.S., Yu H.B., Ji H. (2020). Energy response and fatty acid metabolism in *Onychostoma macrolepis* exposed to low-temperature stress. J. Therm. Biol..

[68] Liu S., Tian F., Qi D., Qi H., Wang Y., Xu S., Zhao K. (2023). Physiological, metabolomic, and transcriptomic reveal metabolic pathway alterations in *Gymnocypris przewalskii* due to cold exposure. BMC Genom..

[69] Bi B., Yuan Y., Zhao Y., He M., Song H., Kong L., Gao Y. (2023). Effect of crowding stress on growth performance, the antioxidant system and humoral immunity in hybrid sturgeon. Aquac. Rep..

